# Things Sanitary

**Published:** 1872-07

**Authors:** Ben. H. Pratt


### THINGS SANITARY.

BEN. H. PRATT, A. M.

The writer's lot is cast in a city of considerable note in a neighboring State. It is a large city considering its age. It is also very young —for a city. In it are wide and long avenues, graded and paved, and kept unusually clear and clean. Our city has a good degree of pride in its fine church edifices—its magnificent banking houses—its large and beautiful mercantile establishments—its general facilities for successful trade—its brilliant prospects for the future. It has developed a remarkable degree of enterprise in the way of substantial organizations for securing a health social status. In a word, it is a remarkable growth, considered in all respects, save in one particular, and an important one, probably the most important of all others—its sanitary regulations.

How such a lax condition of affairs ever obtained—why so little attention has been given to the corporate health—what prevents a reform in the sanitary irregularities of our city even now—these are questions so frequently propounded by the more prudent, and never answered satisfactorily, that it seems to be a settled thing, acquiesced in by citizens generally, that we are to have no better rules than the individual caution of each man and woman in the community can make for him and herself.

Just at this particular time—and indeed for some months back—that dreaded and loathsome disease, small-pox, has been holding high carnival in our midst. It need not have been so.—That a few solitary cases should occur is not at all strange. No place is exempt—especially where a large foreign element prevails. But its spread and increase are due to a gross and inexcusable laxity on the part of our city government to take the matter in hand and assume decisive powers in endeavoring to isolate notorious cases of the disease.

It is a fact which none can dispute, that men have lain sick and died in houses as much frequented by neighbors and friends as though nothing of the kind existed. The funerals are conducted in the usual manner, attended by all the friends, relatives and acquaintances of the deceased. The funeral processions of small-pox victims pass through our thronged thoroughfares at any and all times of the day.—Wakes, an institution peculiar to a certain and large class of our community, are attended in the very presence of the corpse, and the disease thus borne and transplanted in every square of the city. The public prints openly set forth individual instances of this, pointing out the culpable parties by name, and yet the authorities do not move, if they move at all, in earnest.—Now and then public feeling reaches such a point of indignation as to call forth a show of interest in the powers that be, and a single policeman is sent to a single residence to make inquiry and put up the sign upon the house. *Cui bono?* the card is torn down as soon as the blue coat disappears around the corner. If there is such a thing as a pest house belonging to the city, I have not seen or heard of it, nor have I heard of a single case having been removed from its customary residence, but once, and that was a forced one, since the family in which the case occurred, put the patient into the street and locked the door upon him, when the tender-hearted policeman took him to the lock up on our principal business street, and there allowed him to get through the disease as well as he might.

At the present moment the wife of my next door neighbor lies sick with varioloid, taken without the shadow of a doubt from a servant girl given to the practice of attending wakes.—We are under the same roof, he occupying one and I the other part of a double house. In this case, the officer whose duty it should be to inquire into matters of this kind, instead of going to the house, came to me at my place of business and made his inquiries there, stating that if care were taken of the case he wouldn't put any "board" on the house. I remarked that it was very kind of him, and felt particulary proud when he stated that if it were any other house he'd have a board up very quickly. It's a satisfaction, you know, to dwell in such close proximity to the recipients of such marked favors and lenenicy at the hands of the star-bearing executors of civic law.

This case is especially pitiable from the fact that the patient is the mother of three very young children, the youngest but a few weeks old, and her isolation is so complete, that her sufferings under the disease are doubly augmented by her yearnings for her little ones, and her fears lest they too may yet come down with the same malady.

I mention this case because of its proximity to my own dwelling, and not because there are not other and worse instances of a similar character within our city limits. It simply proves how much the citizens of any place may suffer from the criminal neglect of its officers to perform their whole duty to their constituents.—There is no excuse for such neglect—no cloak to hide so grievous a sin, and my only object in placing this article in your columns is the hope that perhaps some one may, by being in time advised of the results of negligence here, arouse to a keener sense of the importance of resorting to the very strictest measures that can be devised for completely isolating *every case* of any contagious disease, and thus nip in the bud the misery it may engender.

Where intelligence and prudence becomes the victims of such maladies as are of a contagious character, there can doubtless be no better or surer preventive of the spread of the disease than the caution of the individual would dictate and employ. The better classes of community have that regard for their neighbor's welfare which instinctively calls up every device for confining disease to prescribed limits. With the lower classes, especially of the foreign population, no such delicate sense of duty seems to obtain. Perhaps it arises from their familiarity with it and hence indifference to it. With such, what their own prudence will not effect must be effected by the law, meted out with no mincing hand. Stern measures, where the health and lives of a whole city full are at stake, are necessary, and justifiable, and must be exacted, even to seeming mercilessness.

A Board of Health cannot be too judiciously chosen; nor should they be selected so much for their medical ability and learning, as for their zeal and executive talent—their impartial sense of duty to the whole body corporate, rather than to the man of highest rank with it. To keenly ferret out and expose all contagious disease, and unhesitatingly and promptly have it conveyed to a place of safety, or so isolated that it cannot harm, is the especial province of a Board of Health, and unless it can do this, it is worse than useless, it is a breeder of contagion, in that it does *not* do that which, but for its existence might be done in some other way.

